# A Data-Driven Noise Reduction Method and Its Application for the Enhancement of Stress Wave Signals

**DOI:** 10.1100/2012/353081

**Published:** 2012-11-20

**Authors:** Hai-Lin Feng, Yi-Ming Fang, Xuan-Qi Xiang, Jian Li, Guan-Hui Li

**Affiliations:** School of Information Engineering, Zhejiang A & F University, Zhejiang, Lin'an 311300, China

## Abstract

Ensemble empirical mode decomposition (EEMD) has been recently used to recover a signal from observed noisy data. Typically this is performed by partial reconstruction or thresholding operation. In this paper we describe an efficient noise reduction method. EEMD is used to decompose a signal into several intrinsic mode functions (IMFs). The time intervals between two adjacent zero-crossings within the IMF, called instantaneous half period (IHP), are used as a criterion to detect and classify the noise oscillations. The undesirable waveforms with a larger IHP are set to zero. Furthermore, the optimum threshold in this approach can be derived from the signal itself using the consecutive mean square error (CMSE). The method is fully data driven, and it requires no prior knowledge of the target signals. This method can be verified with the simulative program by using Matlab. The denoising results are proper. In comparison with other EEMD based methods, it is concluded that the means adopted in this paper is suitable to preprocess the stress wave signals in the wood nondestructive testing.

## 1. Introduction 

Recovery of a signal from observed noisy data is usually regarded as an important preprocessing and has been an area of research for decades. Many algorithms for noise reduction have been reported so far in the literature including traditional linear filter, such as Butterworth low pass filter, Wiener filter, and wavelet based thresholding filter [1]. Most of them have been proved to be effective in removing the unwanted components. For example, Hsu et al. succeeded in removing the aliasing on the original step-edge response curve (SRC) caused by the binning of Moire patterns [2]. However, the linear filtering methods are not very effective when the signals contain sharp edges and impulses of short duration [3]. As for wavelet based denoising methods, it's difficult to select the wavelet base, scale, threshold function, and optimal threshold value. 

 In 1998, empirical mode decomposition (EMD) was designed by Wu and Huang primarily for decomposing the nonlinear and nonstationary signals into a series of intrinsic mode functions (IMFs) [4]. The main advantage of EMD is that it depends entirely on the data itself. Consequently, the results preserve the full nonstationarity characteristics of the target signals. Seen in this light, the EMD method is superior to the wavelet analysis approach, where the basic functions are fixed and, thus, do not necessarily match all real signals [3]. The property of EMD to behave as a dyadic filter bank resembling those involved in wavelets [5] has been useful in signal denoising and received more and more attention [3, 5–11]. 

 However, the EMDalgorithm may encounter the problem ofmode mixingwhen a signal contains intermittency. Therefore the ensemble empirical mode decomposition (EEMD) was introduced [12]. The algorithm defines the IMF set for an ensemble of trials, each one obtained by applying EMD to the signal of interest with added independent identically distributed white noise of the same standard deviation. Taking into account properties of the white noise, the problem of mode mixing can be overcome [13]. Owing to the impressive performance, EEMD has been used to address several problems in the field of science and engineering. It was employed to calculate the residual signal with the purpose of detecting localized gearbox faults of damage at an early stage [14]. In [15], the authors used it to predict the short term passenger flow with back-propagation neural networks (BPN). Zhang and Xie decomposed the impact echo signals into different spectral composition for defect signal extraction [16].

 As a more robust and noise-assisted version of EMD, EEMD can be used as an alternate in EMD based denoising methods. Furthermore, the use of the EEMD process as a filter and its comparisons with the EMD method have just recently been studied in [17, 18]. An improved filtering performance can be achieved by EEMD than EMD with suitable added noise and sufficient trial number. In our earlier paper, we proposed a signal denoising strategy using EEMD combined with the instantaneous half period (IHP) to restore stress wave signal from observed raw data [19].

 In this paper, we investigated an improvement based on the above method. The main contribution of this paper is that, utilizing the consecutive mean square error (CMSE), we can determine the optimum threshold adaptively. The method can work well on condition that no prior knowledge is required. The whole procedure is fully data driven.

## 2. Conventional EEMD Based Filtering **** Approach

### 2.1. EMD Algorithm

The EMD algorithm can be described as follows [4].

1. Extract all the local maxima and minima of *x*(*k*).
2. Form the upper and lower envelop by cubic spline interpolation of the extrema point developed in step (1).
3. Calculate the mean function of the upper and lower envelop, *m*_1_(*k*).
4. Let *h*_1_(*k*) = *x*(*k*) − *m*_1_(*k*). If *h*_1_(*k*) is a zero-mean process, then the iteration stops and *h*_1_(*k*) is an IMF1, named *c*_1_(*k*), else go to step (1).
5. Define *r*(*k*) = *x*(*k*) − *c*_1_(*k*).
6. If *r*(*k*) still has least 2 extrema then go to step (1) else decomposition process is finished.

At the end of the procedure, we have a residue *r*(*k*) and a collection of *n* IMF, named from *c*_1_(*k*) to *c*_*n*_(*k*). The original signal can be represented as

(1) $$\begin{array}{c} x\left(k\right)=\sum_{i=1}^{n}c_{i}\left(k\right)+r\left(k\right). \end{array}$$

### 2.2. EEMD Algorithm

The steps for EEMD are as follows [12].

(1) Initialize the number of ensemble *J*, the amplitude of the added white noise, and *j* = 1.
(2) Add a white noise series to the targeted signal,  *x*_*j*_(*k*) = *x*(*k*) + *n*_*j*_(*k*).
(3) Apply EMD to the noise-added signal *x*_*j*_(*k*) to derive a set of IMFs *c*_*i*,*j*_(*k*) (*i* = 1,2,…, *n*) and residues *r*_*j*_(*k*), where *c*_*i*,*j*_(*k*) denotes the *i*th IMF of the *j*th trial, and *n* is the number of IMFs.
(4) Repeat steps (1) and (2) until *j* > *J*.
(5) Average over the ensemble to obtain the final IMF of decompositions as the desired output:
  (2) $$\begin{array}{c} {\overset{-}{c}}_{i}\left(k\right)=\frac{1}{J}\sum_{j=1}^{J}c_{i,j}\left(k\right)\;\left(i=1,2,\ldots ,n\right),\\ \overset{-}{r}\left(k\right)=\frac{1}{J}\sum_{j=1}^{J}r_{j}\left(k\right). \end{array}$$

Just as the EMD method, the given signal, *x*(*k*) can be reconstructed according to the following equation:

(3) $$\begin{array}{c} x\left(k\right)=\sum_{i=1}^{n}{\overset{-}{c}}_{i}\left(k\right)+\overset{-}{r}\left(k\right). \end{array}$$

In contrast with EMD, EEMD skillfully eliminates the mode mixing phenomenon and the results obtained by EEMD reflect the nature of signals more accurately [20]. In spite of a heavy computational load, it is still suitable for getting better performance [17, 18]. So the EEMD algorithm is utilized in this study. Furthermore, to illustrate the performance of the present approach, we restricted ourselves to the EEMD method which was used as the mean to decompose the signal in both EMD based method and EEMD based method involved in this study.

### 2.3. EEMD Based Filtering Approach

**Figure 1:**
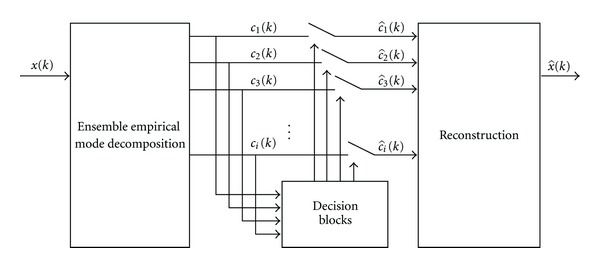
Conceptual model of noise reduction method using EEMD.

A conceptual model of EEMD based filtering approach is depicted in Figure 1. It consists of three steps. First, noisy signal is adaptively decomposed into IMFs by means of EEMD algorithm. In the next stage, these IMFs are classified and detected in terms of their different properties under certain criteria and those undesirable IMFs are thereby removed by switching corresponding switches “off”, that is, setting the values of the corresponding *c*_*i*_(*k*) to zeros, and will not be used in the signal reconstruction. Finally, the recovered signal is reconstructed with only a few IMFs which are signal dominated. Thus it is reasonable to assume that a full restoration is possible by this approach if enough information is available on the characteristics of underlying signals, depending on the selection and rejection of the IMFs.

The second step is typically performed by two operations. Based on this, the existing EEMD based method can be divided into two categories: EEMD based thresholding filter [6, 8, 21] or EEMD based low pass filter [3, 17, 21]. The former method reconstructs the signals with all the IMFs, which use the previous threshold as in wavelet analysis. Because most of the important structures of the signal are often concentrated on lower frequency components (high order IMFs), and they generally decrease towards the high frequency modes (low order IMFs), the noise power can be reduced significantly by adding a suitable threshold on the high frequency modes. However, when applying the threshold on the high order IMFs with little or no noise, the main features of the original signal may be lost or changed accordingly. 

 The latter method, EEMD-based low pass filter, was developed based on the assumption that the IMFs derived by EEMD can only be divided into two classes: noise-only IMFs and signal-only IMFs. Accordingly, it is feasible to use a criterion to classify and remove the noise-only IMFs, which leads to the result that the signal-only IMFs are partially reconstructed. However, noises are usually distributed over all IMFs. Thus the low pass filter based on EEMD will remove the high frequency components of both the noise and the signal, and the low frequency components of noise also remain.

## 3. The Data-Driven IHP (DIHP) Approach

In our presented method, the *i*th IMF, *i* = 1,2,…, *n*, can be denoted as *c*_*i*_(*k*), where *n* is the number of IMFs. Accordingly, we can use mathematical operations to locate the zero-crossings of *c*_*i*_(*k*). The symbol *ZP*_*i*_^*j*^ is used to define the *j*th zero-crossing of the *i*th IMF correspondingly. Moreover, the time when the *j*th zero-crossing of the *i*th IMF emerges is defined as *τ*_*i*_^*j*^. As a result, the time interval between *ZP*_*i*_^*j*+1^ and *ZP*_*i*_^*j*^ can be treated as the half period of an oscillation, which is used in our method as a criterion. Considering the half periods may be different from each other, we define it as IHP and the formula can be expressed as

(4) $$\begin{array}{c} T_{i}^{j}=\tau_{i}^{j+1}-\tau_{i}^{j}. \end{array}$$

Usually, the signal structures are corresponding to the slow time variation of data. Besides, the frequency of the signal is often lower than that of the noise structures [3]. Consequently, it can be supposed that the IHP of a signal dominated oscillation is longer than the IHP of a noise dominated oscillation. Based on this assumption, the symbol thr is introduced to be a threshold, which allows us to retrieve the most important structures of the signal from its noisy version. If the IHP is bigger than thr, the waveforms between the two adjacent zero-crossings will be considered as signal dominated oscillations and be retained, whereas the waveforms with smaller LHP will be treated as noise dominated oscillations and be set to zeros. This process can be described as

(5) $$\begin{array}{c} {\hat{c}}_{i}\left(k\right)=\{\begin{array}{cc} c_{i}\left(k\right), & T_{i}^{j}\geq \text{thr},\\ 0, & \text{others}, \end{array}\;\;ZP_{i}^{j}<k\leq ZP_{i}^{j+1}. \end{array}$$

Finally a reconstruction process of projecting the restored IMF, ${\hat{c}}_{i}(k)$, back onto the filtered signals is done as follows:

(6) $$\begin{array}{c} \hat{x}\left(k\right)=\sum_{i=1}^{n}{\hat{c}}_{i}\left(k\right)+\hat{r}\left(k\right). \end{array}$$

Note that the filtering effect is related to the value of thr. A large thr would result in oversmoothing of the target signal, thus removing some low-frequency oscillations while these oscillations are signal dominated. Moreover a small thr might not be able to remove the artifacts, hence resulting in a signal of relatively low quality. We have earlier reported a solution based on the maximum frequency and a constant coefficient which can be determined with experience [19]. In this paper, we concern the method under the condition that no prior knowledge about the target signal is required. It is a very common problem because the prior knowledge is unavailable or can be obtained with high cost in many applications.

 In general, the aim of the filtering is to find an approximation reconstructed signal $\hat{x}(k)$ from the observed signal *x*(*k*) with minimum errors, that is, with lower distortion measures such as mean square error (MSE), mean absolute error (MAE), or mean square difference (MAD). Unfortunately, these measures can not be calculated because the original signal is unknown in practice. Boudraa proposed a criterion, called CMSE, based on the squared Euclidean distance between two consecutive reconstructions of the signal. It does not require any knowledge of the target signal and is a fully data-driven approach. In this study, we attempted to find the optimum threshold thr_opt_ by minimizing the cost function CMSE

(7) $$\begin{array}{c} {\text{thr}}_{\text{opt}}=\arg\;\min\left\{\text{CMSE}\left({\hat{x}}_{m}\left(k\right),{\hat{x}}_{m+1}\left(k\right)\right)\right\}, \end{array}$$

The CMSE is defined as follows:

(8) $$\begin{array}{c} \text{CMSE}\left({\hat{x}}_{m}\left(k\right),{\hat{x}}_{m+1}\left(k\right)\right)≜\frac{1}{L}\sum_{k=0}^{L-1}{\left[{\hat{x}}_{m}\left(k\right)-{\hat{x}}_{m+1}\left(k\right)\right]}^{2}, \end{array}$$

where ${\hat{x}}_{m}(k)$ and ${\hat{x}}_{m+1}(k)$ are signals that are reconstructed using the thr = *m* and thr = *m* + 1, respectively.

## 4. Experiments and Results

### 4.1. Stress Wave Signals Description

During the past few decades, many advanced signal processing algorithms have been utilized to analysis the stress wave signal for the wood nondestructive testing, such as spectral analysis [22], wavelet analysis [23]. Unfortunately, no developed method or system is used worldwide until now due to the difficulty to extract useful information directly from the raw signals. The collected signals can be viewed as the result of multiple interferences and reflections of these two waves fitting the boundary conditions, which interfered with the stress wave information identification [24]. Therefore, noise reduction is a necessary step for any stress wave based wood test technique to pave the way for further discovery in physics and nature [19]. 

**Figure 2:**
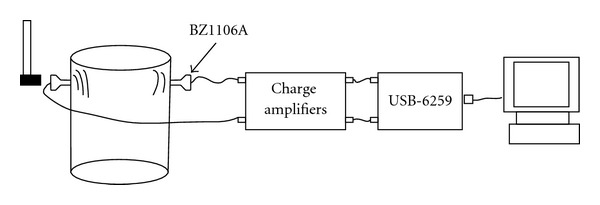
The self-developed signal collection system.

**Figure 3:**
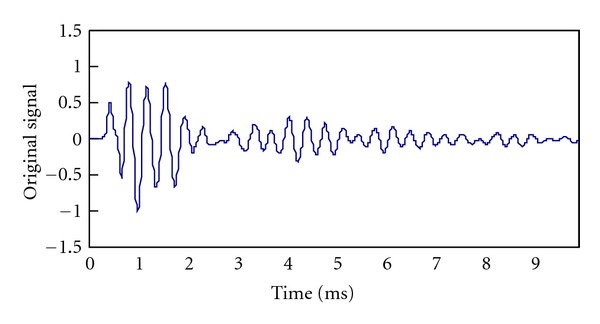
A typical stress wave signal used in this study.

Figure 2 illustrates the signal acquisition system which was used to collected stress wave signals in this study. We selected a typical *Cinnamomum camphora* trunk with a diameter of 27 cm as the sample. The piezoelectric receivers BZ1106A, coupled with the matching charge amplifiers, from Beidaihe Institute of Electrical Automation were employed. The DAQ instrument was USB-6259 card manufactured by National Instruments. A typical signal recorded is shown in Figure 3. The sampling rate is 100 KHz and the duration is 10 ms.

**Figure 4:**
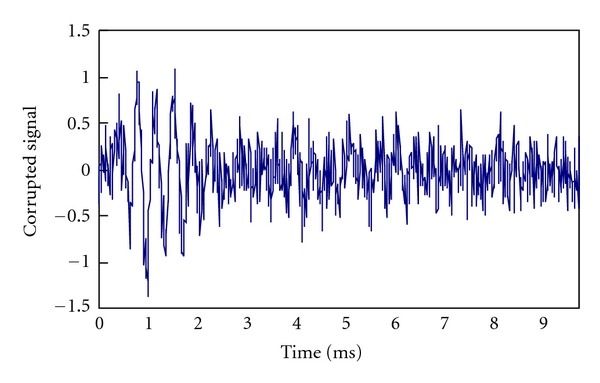
The corrupted version of the signal shown in Figure 3.

Gaussian White Noise is added as noise with zero mean. A noisy version of the signal shown in Figure 3 is depicted in Figure 4. The SNR is 0 dB.

### 4.2. The Performance Evaluation of Finding the Optimum Threshold

**Figure 5:**
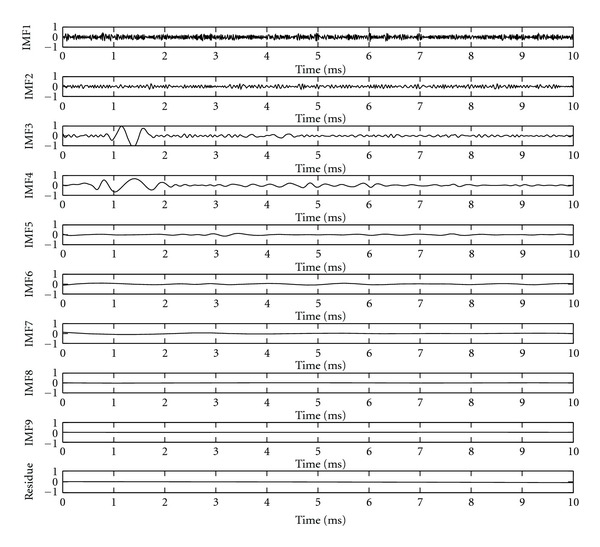
The decomposition result with EEMD.

In this section, we should validate the presented method's ability of finding the optimum threshold thr_opt_. Firstly, the signal shown in Figure 4 is decomposed into several monotonic components (IMFs) of distinct time scales as shown in Figure 5 using EEMD method. The parameters used to run the EEMD are the number of ensemble, *M*, and amplitude of the added white noise, *k*, which is set to 100 and 0.2 time standard deviation [12]. Here, we can see fast oscillations in the lower-order IMFs, and slow oscillations as the order of components increases.

**Figure 6:**
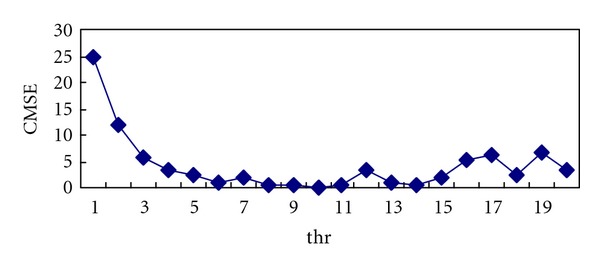
CMSE versus *m*.

Then CMSE was calculated under different value of *m*.  Figure 6  shows  the variety of CMSE when the *m* changed from 1 to 20. It is obviously suggested in the figure that the CMSE reaches the minimum value when *m* gets the value 10. 

**Figure 7:**
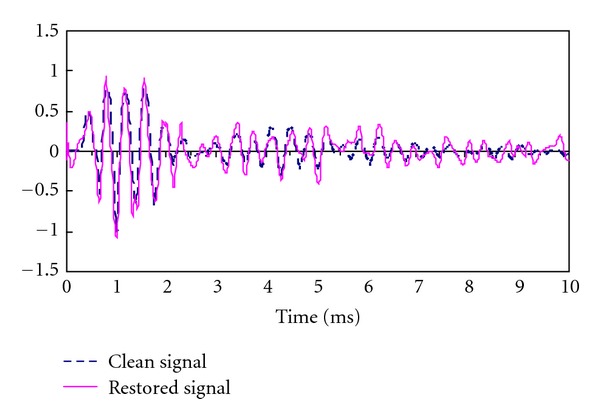
The denoised stress wave signal using proposed method.

After that, the thresholding operation as described in (5) was performed and followed by the reconstruction process defined in (6).  Figure 7 shows the results. As a comparable result, the purified signals are also given in the figure. It is obviously obtained that the noise signals have been restrained notably and the recovered signals come close to the original signals.

### 4.3. Comparison with Other EEMD-Based Methods

To verify the efficiency of the presented denoised method under different levels of noises, we designed an experiment to compare the results in different SNR with other two EEMD algorithms. The signals with distinct SNR were first generated and the SNR ranged from −5 to 15. The MSE was still selected as the criterion of evaluation. The definition of MSE is described as follows:

(9) $$\begin{array}{c} \text{MSE}=\frac{1}{L}\sum_{k=0}^{L-1}{\left[s(k)-\hat{x}(k)\right]}^{2}, \end{array}$$

where *s*(*k*) and $\hat{x}(k)$ denote the values of the original signal and restored signal, and *L* is the length.

**Figure 8:**
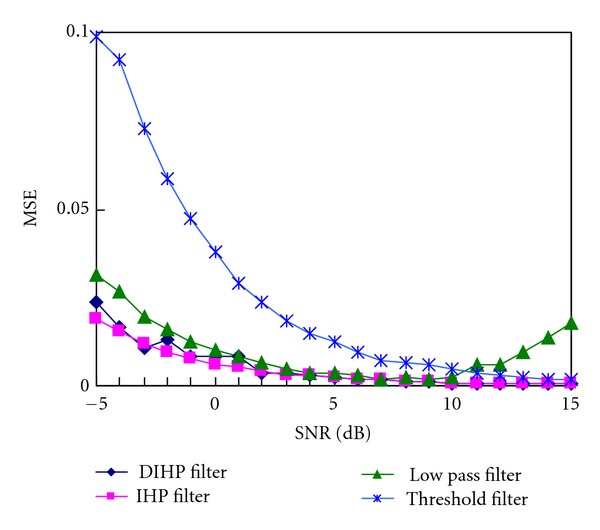
MSE obtained with different noise levels by proposed method, IHP filter, EEMD-based low pass filter, and EEMD-based thresholding filter.

The experimental results are shown in Figure 8. The MSE in the figure was gained by computing the average value of 10-time repeat. For comparison, the results of EEMD based low pass filter, EEMD based thresholding filter, and IHP filter [19] were shown in Figure 8. It is evident that the proposed DIHP filter has given a better performance compared to the EEMD based low pass filter and the EEMD based thresholding filter. What should be emphasized here is that the result of DIHP filter is close to that of IHP. It is capable of producing better noise-removal results even in cases where the signal quality is low (SNR value is −5 dB). This means that the method is effective for very noisy signals. It implies we can get the same effect on conditions that any prior knowledge of the target signals is. The proposed method is fully data driven.

## 5. Conclusions

A new technique for the signal enhancement has been proposed and developed. Simulations results of the synthesized signals have expressed the effectiveness of the new algorithm. The technique differs from many EEMD based algorithms as it uses IHP to detect and classify the noise oscillations and utilizes CMSE to compute the optimum threshold adaptively. The filtering method is a fully data-driven approach. As a result, the proposed technique has the ability to be used as a preprocessing step for computerized wood nondestructive test using the stress wave technique.
